# Sodium Salt of Partially Carboxymethylated Sodium Alginate-Graft-Poly(Acrylonitrile): II Superabsorbency, Salt Sensitivity and Swelling Kinetics of Hydrogel, H-Na-PCMSA-g-PAN

**DOI:** 10.3390/gels9050407

**Published:** 2023-05-12

**Authors:** Jignesh Trivedi, Arvind Chourasia

**Affiliations:** 1 Post Graduate Department of Chemistry, Sardar Patel University, Vallabh Vidyanagar 388120, Gujarat State, India; 2 Tridev Resins (India) Pvt. Ltd. 136/E-1, II Phase, G.I.D.C., Vapi 396195, Gujarat State, India

**Keywords:** sodium salt of partially carboxymethylated sodium alginate, superabsorbent hydrogel, swelling behavior, salt sensitivity, swelling kinetics

## Abstract

The water absorption measurements of a novel superabsorbent anionic hydrogel, H-Na-PCMSA-g-PAN, has been reported first time in water with a poor conductivity, 0.15 M saline (NaCl, CaCl_2,_ and AlCl_3_) solutions, and simulated urine (SU) solutions at various times. The hydrogel has been prepared by the saponification of the graft copolymer, Na-PCMSA-g-PAN (%G = 316.53, %GE = 99.31). Results indicated that as compared to the swelling capacity values evaluated in water with a poor conductivity, the ability of the hydrogel to swell in various saline solutions with the same concentration is significantly reduced at all different durations. The swelling tends to be Na^+^ > Ca^2+^ > Al^3+^ at the same saline concentration in the solution. Studies of the absorbency in various aqueous saline (NaCl) solutions also revealed that the swelling capacity decreased as the ionic strength of the swelling medium rose, which is consistent with the experimental results and Flory’s equation. Furthermore, the experimental results strongly suggested that second-order kinetics governs the swelling process of the hydrogel in various swelling media. The swelling characteristics and equilibrium water contents for the hydrogel in various swelling media have also been researched. The hydrogel samples have been successfully characterized by FTIR to show the change in chemical environment to COO^−^ and CONH_2_ groups after swelling in different swelling media. The samples have also been characterized by SEM technique.

## 1. Introduction

Among commonly used types of hydrogels, much interest has been shown in the development of the synthesis of biopolymers-based superabsorbent hydrogels [1,2,3,4,5] because of their biocompatibility, hydrophilicity, biodegradability, and non-toxicity. These hydrogels are three-dimensional hydrophilic networks capable of absorbing large quantities of water, saline, or physiological solutions [2]. The superabsorbent hydrogels are widely used in many fields such as agricultural and horticultural, disposable diapers, feminine napkins, pharmaceuticals, and medical applications [1,2,3,4,5,6] as they possess excellent characteristics such as swelling, mechanical, permeation, surface, and optical properties. Several methods for the synthesis of hydrogels have been reported i.e., based on a chemical method using crosslinker, a physical method using a complexing agent, and irradiation methods using gamma-ray irradiation or electron beam [1,6,7]. However, free radical graft copolymerization of vinyl monomers onto biopolymer backbone followed by crosslinking via saponification of the nitrile groups of the produced graft copolymer is also a favorable and successful method [8,9,10,11,12,13].

Sodium Alginate (SA) is an anionic biopolymer, which is derived from brown seaweeds, and is composed of poly-β-1, 4-D-mannuronic acid (M Units) and α-1, 4-L-guluronic acid (G Units) in varying proportions through 1–4 linkages. SA is a biocompatible, biodegradable, non-toxic, chelatable, and gelablebiological macromolecule and it is suitable for chemical modification [14]. A comprehensive literature survey reveals that there are some reports on the preparation of superabsorbent hydrogels based on sodium alginate [15,16,17,18,19,20]. 

However, SA hydrogels suffer from low water absorbency and poor mechanical properties. In view of this, attempts have been made to synthesize superabsorbent hydrogels through saponification of sodium alginate-graft polymethyl acrylamide [21], sodium alginate-graft-polyacrylamide [22] and alginate-polyacrylonitrile physical mixture [23]. Recently, Lin et al. [24] created hydrogels by polymerizing etherified sodium alginate, sodium acrylic acid, and polyvinyl alcohol in aqueous solution, and they assessed how responsive the hydrogels were to pH and saline. However, the review of the literature indicates that there is no information on the creation of a superabsorbent hydrogel by saponification of the graft copolymer, Na-PCMSA-g-PAN, and its measurements on super absorbency, salt sensitivity, and swelling kinetics. 

In continuation of the first part of this study [25], we have thoroughly examined the swelling pattern of a novel salt-sensitive superabsorbent hydrogel, H-Na-PCMSA-g-PAN, synthesized upon saponification of the optimally prepared Na-PCMSA-g-PAN, in various swelling media. In order to explore the studies of the swelling kinetics of the hydrogel, the water absorption findings in various swelling media are analyzed. The values of the swelling characteristics of the hydrogel in various swelling media are also presented. 

## 2. Results and Discussion

Superabsorbent hydrogels (SHs) are recognized for their swelling capacity, which can be assessed by their absorption mechanism, which is in turn brought on by the diffusion process signifying the affinity between the polymer networks and the external fluids. According to the Donnan Equilibrium hypothesis [26], the equilibrium between the polymer grid’s flexibility and the osmotic pressure inside the expanding system determines how much swelling will occur. The hydrogel network’s internal structure and the surrounding mixtures have different mobile ion concentrations, which causes an elevation of osmotic pressure. In this case, the grid of polymers attachment of ionizable carboxylate groups (anionic sites), Na-PCMSA, is what is responsible for the uneven distribution of ions.

### 2.1. Water Absorbency Measurements

**Figure 1:**
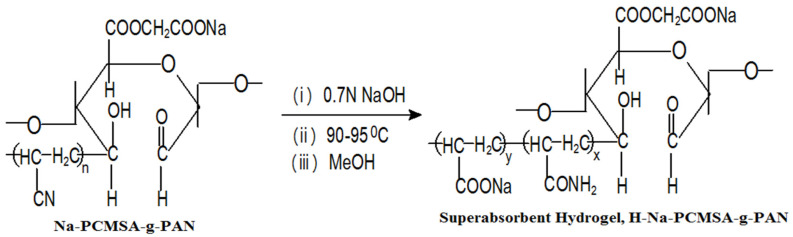
Saponification of Na-PCMSA-g-PAN to create superabsorbent hydrogel.

**Figure 2:**
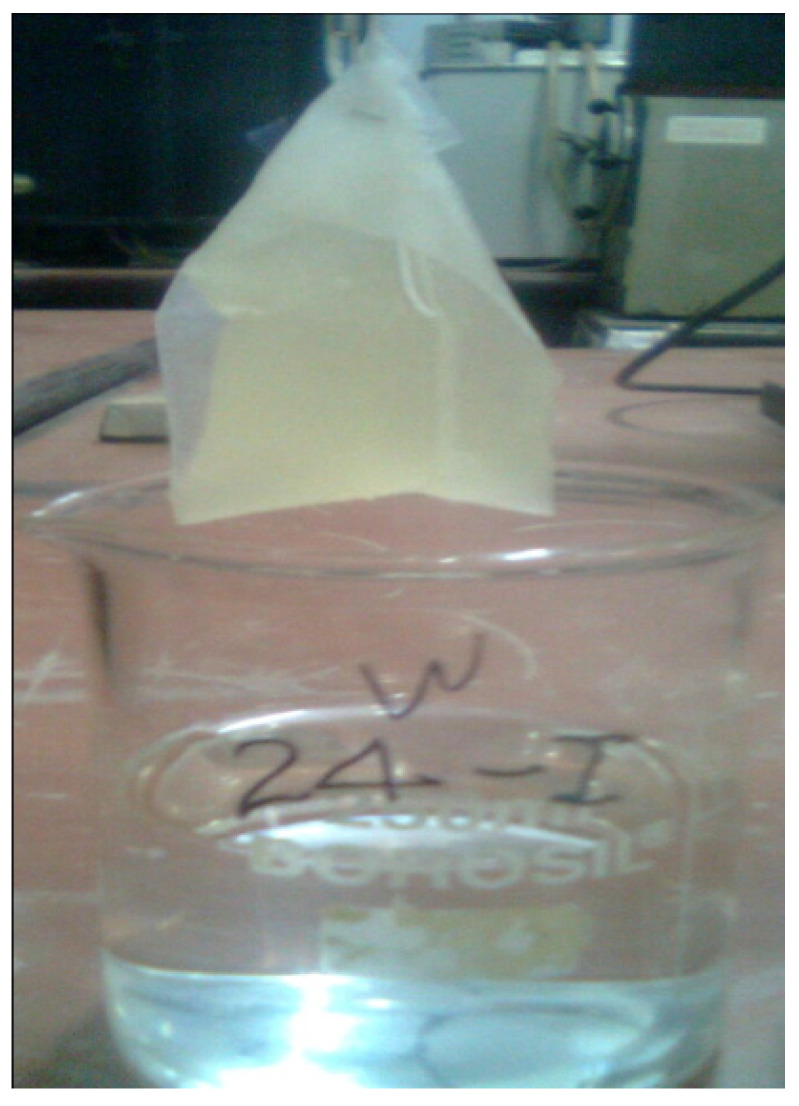
Photograph of the superabsorbent hydrogel after 24 h of its immersion in water with a poor conductivity.

Figure 1 depicts the H-Na-PCMSA-g-PAN superabsorbent hydrogel structure created in the current work. However, its typical photograph after 24 h of immersion in water with a poor conductivity at room temperature is represented in Figure 2. 

**Figure 3:**
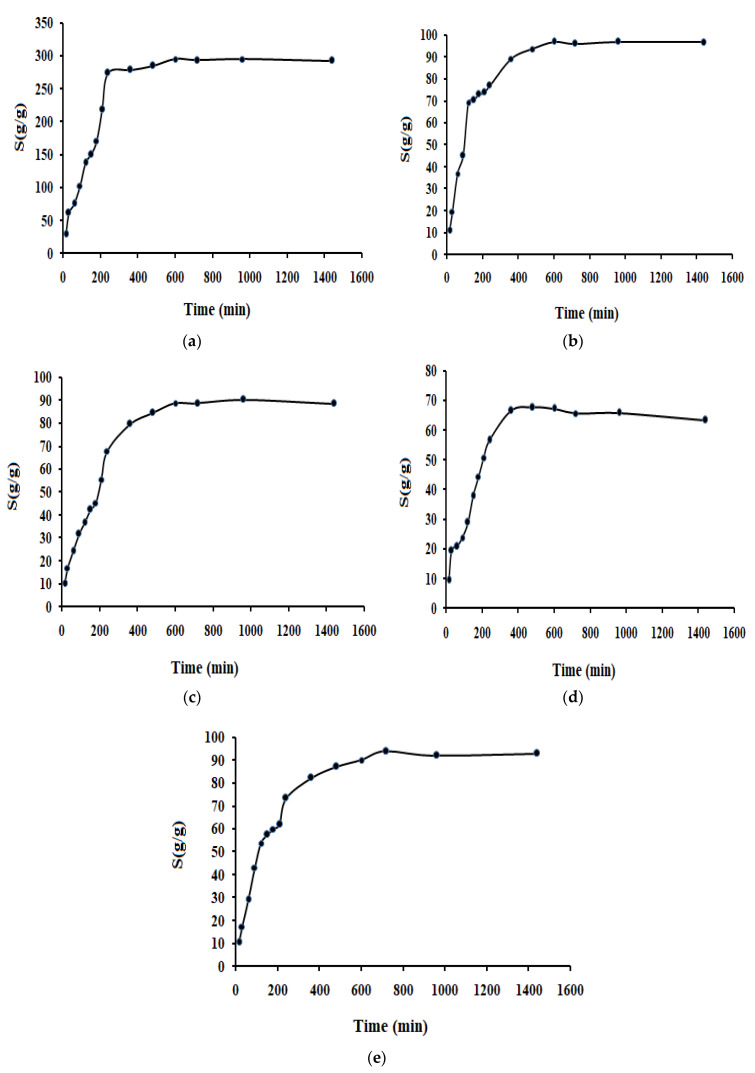
Dynamic swelling curves for the superabsorbent hydrogel, in (**a**) water with a poor conductivity; (**b**) 0.15 M NaCl solution; (**c**) 0.15 M CaCl_2_ solution; (**d**) 0.15 M AlCl_3_ solution and (**e**) 0.15 M SU solution.

The anionic superabsorbent hydrogel, H-Na-PCMSA-g-PAN, demonstrates swelling behavior in Figure 3a as a function of time in low-conductivity water. It becomes evident from the results that the rate of water uptake sharply increases within about 200 min. and then begins to level off. The hydrophilic groups, including carboxylates and carboxamides, present in the hydrogel, absorb the penetrating water by forming hydrogen bonds with it. Both the osmotic pressure difference between the hydrogel and the external solution and the repulsion of anionic hydrophilic groups inside the network are responsible for the swelling. However, even after 30 min, the hydrogel quicklyabsorbs 61.29 g/g gel water. The hydrogel is absorbing water at a rate of about 1.97 g/g min at this moment (30 min). It is found that 600 min are needed to obtain the equilibrium swelling capacity.

### 2.2. Swelling in Salt Solutions

The ionic superabsorbent’s swelling phenomenon is significantly influenced by the properties of external solutions, including their charge valences and ionic strength. In the current study, we examined how the super absorbent hydrogel, swelled in both synthetic urine (SU) solution and saline solutions with the same concentrations (0.15 M) of NaCl, CaCl_2_, and AlCl_3_. The results are displayed in Figure 3b–e. The absorbency was significantly lower in the presence of all saline solutions than it was in water with a poor conductivity [cf. Figure 3b–e]. The development of osmotic pressure as a result of the uneven distribution of ions in the swelling media and the polymer network is a possible explanation for this phenomenon. It is believed that a semi-permeable membrane separates the immobile ions linked to the polymer network from the external solution. The largest osmotic pressure develops when the hydrogel is submerged in water with a poor conductivity, which results in the maximum swelling (295.31 g/g) [cf. Figure 3a]. However, it is discovered that the ability of the hydrogel to swell is significantly reduced in all saline solutions (NaCl or CaCl_2_ or AlCl_3_ or SU), which may have been brought on by an alteration in the ionic pressure variance among the swelling medium and polymeric gel network.

**Figure 4:**
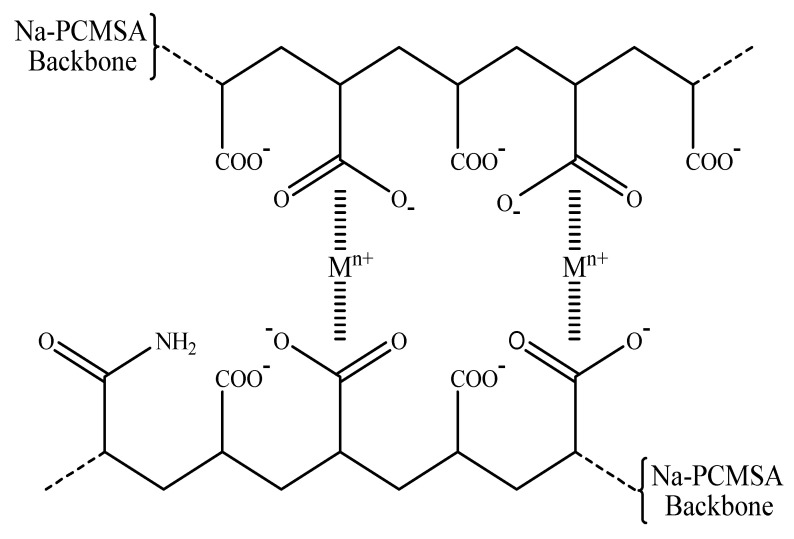
Complexing capacity of H-Na-PCMSA-g-PAN, considering the relationship between the anionic groups of the hydrogel and multivalent metal cations (Ca^2+^ and Al^3+^).

Figure 3b–e results can also be used to explain how different cations that share a common anion affect the absorption capacity of the superabsorbent hydrogel, H-Na-PCMSA-g-PAN. It was discernible from these figures that, in solutions with the same concentration (0.15 M), as the metal cation’s charge enhances, the hydrogel’s absorption capacity decreases. This may be explained by the fact that in multivalent cationic solutions, complexes between multivalent cations and hydrophilic groups (carboxylate or carboxamide groups) lead to the formation of “ionic cross-links” in the superabsorbent networks (Figure 4), increasing the cross-linked density and lowering the swelling capacity. Because of this, the synthesized superabsorbent hydrogel’s swelling capacity is in the following order: NaCl > CaCl_2_ > AlCl_3_ [cf. Figure 3b–d] [27].

Because “ionic-crosslinking” primarily takes place at the particle’s surface, the hydrogels, in this case, were discovered to be extremely hard and rubbery to the touch when they swelled in calcium chloride and aluminium chloride solutions, which prevented them from swelling properly. This is due to the ionic crosslinking phenomenon primarily arising at the surface of the particles. The hydrogel particles, on the contrary, were observed to be swollen in sodium chloride and water and to possess reduced gel strength towards the touch. These findings concur will those mentioned in the literature [13,28], as well.

Flory’s equation can be used to express the relationship between ionic strength and water absorbency [29]:

(1) $$Q^{5/3}\;≅\;\frac{{\left(i/2V_{u}I^{1/2}\right)}^{2}\;+\;(1/2\;-\;\chi_{1})/V_{1}}{V_{e}/V_{0}}$$

where Q is water absorbency or degree of swelling, i/Vu is the charge density of polymer, I is the ionic strength of a solution, $(1/2\;-\;\chi_{1})/V_{1}$ is the polymer-solvent affinity, and $V_{e}/V_{0}$ is the cross-linked density. Thus, Q depends on the cross-linked density, ionic osmotic pressure, and water affinity of the hydrogel. The first and second variables in the numerator are associated with forces that support swelling behavior promotion. 

**Figure 5:**
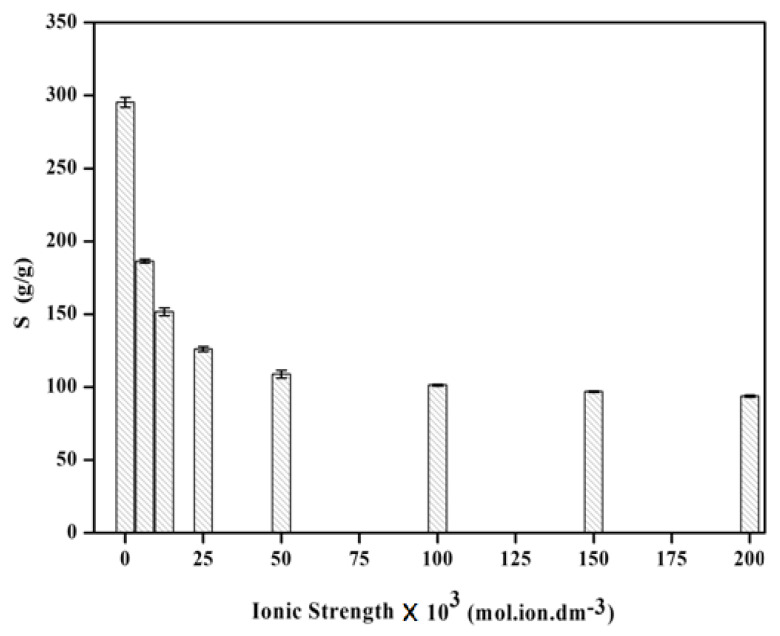
Swelling capacity variation of H-Na-PCMSA-g-PAN hydrogel sample in NaCl solutions having different ionic strengths.

Figure 5 illustrates the ionic strength’s impact (concentration) on the inflammation ability of the superabsorbent hydrogel. The experimental results are consistent with Flory’s equation because it can be seen that the hydrogel’s ability to swell reduces as the ionic strength of different external NaCl solutions increases. However, the decrease in the swelling capacity is rapid initially and then gradual at higher NaCl concentrations. The figure also shows that altering the NaCl content above 0.15 M has no discernable impact on the capacity for absorption. The “charge screening effect” in the saline solutions can be used to explain the results of Figure 5. Additionally, the extra cations lead to imperfect anion-anion electrostatic repulsion reducing the osmosis index differential that exists among the external solution and the network of hydrogel and its ability to swell.

From the results of Figure 3b–e, the values of the dimensionless saline sensitivity factor (f) were calculated using the following Equation (2) [13], and the outcomes are tabularized in Table 1.

(2) f=1−(Ssalt/Swater)

where Ssalt and Swater are, respectively, the swelling capacity values in a given saline solution and water with a poor conductivity.

On the basis of the values of f (Table 1), it can be concluded that the values of f are found to be higher in 0.15 M SU (containing multivalent cations like Mg^2+^, Ca^2+^, and Na^+^), CaCl_2_ and AlCl_3_ solutions in comparison with NaCl solution. These findings may be explained by the hydrogel’s degree of cross-linking, which is shown by a reduction in the binding of trivalent to monovalent cations to its functional groups ($-COO^{-}$). The average f values of the superabsorbent hydrogel are, therefore, observed to be greater in 0.15 M AlCl_3_, CaCl_2_, and SU solutions compared to the NaCl solution due to ionic cross-linking by multivalent cations. In other words, when the swelling media is 0.15 M AlCl_3_, CaCl_2_, and SU solutions, the greater average saline sensitivity values are due to the high charge screening effect of the hydrogel. In the literature, similar outcomes are also observed [30].

### 2.3. Kinetic Analysis

#### Swelling Kinetics in Swelling Media

To account for the given experimental data, both the pseudo-first-order [31,32] and pseudo-second-order [33,34] models were considered to examine the mechanism. However, the pseudo-second-order swelling kinetic model had been employed because the experimental data were not appropriate for the pseudo-first-order swelling kinetic model satisfactorily and accordingly the swelling rate can be described as at any time by:

(3) $$\frac{dS}{dt}\;=\;k_{S}{\left(Seq-S\right)}^{2}$$

where S_eq_, S and $k_{S}$ represent the equilibrium swelling (theoretical), swelling at any time, and swelling rate constant respectively. Equation (3) is integrated above the boundaries S = S_0_, t = t_0_ and S = S at t=t provides

(4) t/S = A+ Bt

In which B = 1/S_eq_ states the inverse of the maximum or equilibrium swelling, A = 1/$k_{S}.S_{eq}^{2}$ represents the reciprocal of the initial swelling rate of the hydrogel $\left(r_{i}\right)$ and $k_{S}$ denotes the swelling rate constant.

**Figure 6:**
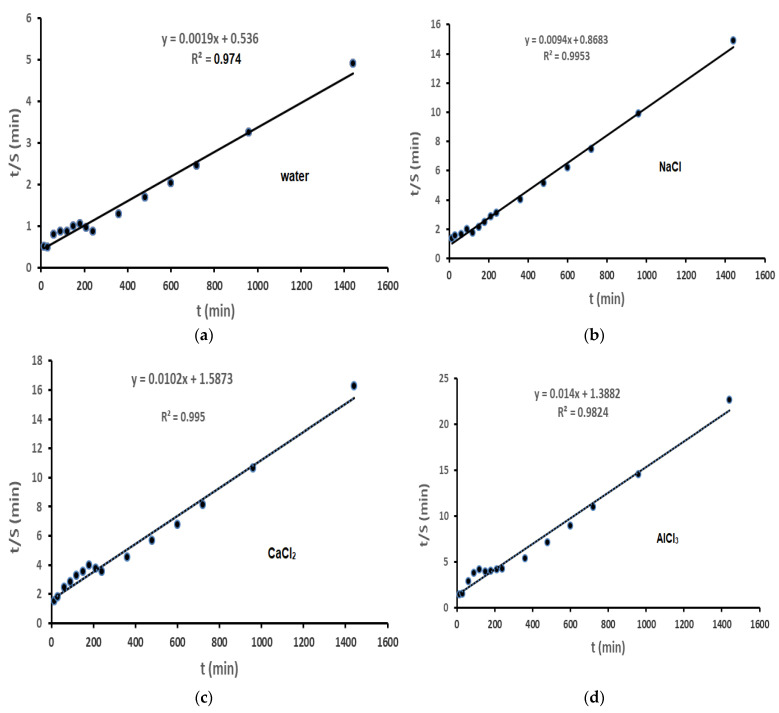

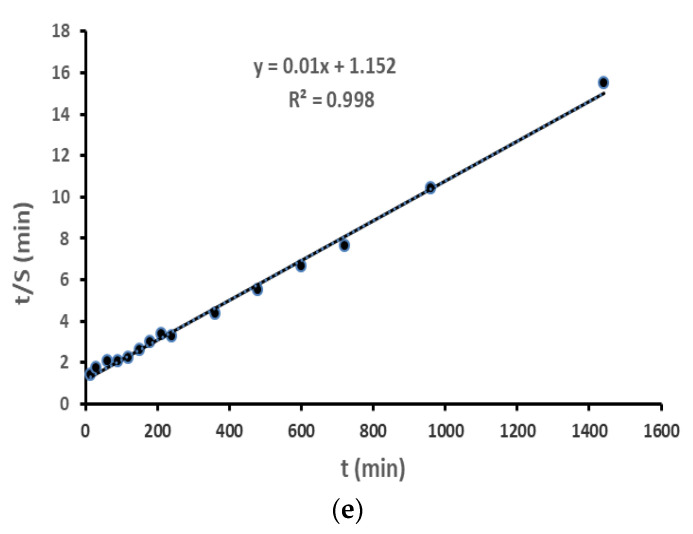
Plots of t/S versus t for H-Na-PCMSA-g-PAN, in (**a**) water with a poor conductivity; (**b**) 0.15 M NaCl solution; (**c**) 0.15 M CaCl_2_ solution; (**d**) 0.15 M AlCl_3_ solution; (**e**) 0.15 M SU solution.

Figure 6a–e followed by Equation (4) displays the typical plots of t/S versus t obtained for super absorbent hydrogel in water with a poor conductivity, NaCl, CaCl_2_, AlCl_3,_ and SU (0.15 M) solutions. Given that the plots had a strong linear correlation coefficient and were confirmed to be linear (Table 2), the hydrogel behaves in ways reliable with the pseudo-second-order kinetic model while swelling in various swelling media. The initial rate of swelling $\left(r_{i}\right)$ was found to be higher when water with a poor conductivity was used as the inflammatory medium, measuring 1.87 [(g water/g gel) min], when compared to the outcomes obtained for other media, such as different salt solutions (0.15 M) and simulated urine (SU). In addition, it is observed that the value $r_{i}$ decreases as ionic strength (salt type) of the swelling media increases, except for the AlCl_3_ solution.

However, the drop of the osmosis index differential among the gel network and the corresponding solution is responsible for the observed depletion in the initial swelling rate $\left(r_{i}\right)$ as the salt solution’s ionic strength (type of salt) increases, showing that the experimental results are consistent with the kinetic model of second-order swelling (Table 2). The experimental equilibrium swelling capacity values for various media together with the related time needed to reach these capacity values are shown in Table 2. Additionally, the hydrogel’s equilibrium water content (EWC%) values in various swelling media were calculated following Equation (6) and are also incorporated in Table 2.

### 2.4. Characterization

#### 2.4.1. Infrared Spectroscopy

Infrared spectroscopy was employed not only to confirm the structural differences of the graft copolymer and the hydrogel but also to show the change in the chemical environment to $-COO^{-}$ and −CONH_2_ groups after swelling of the hydrogel in various saline solutions and water with a poor conductivity.

**Figure 7:**
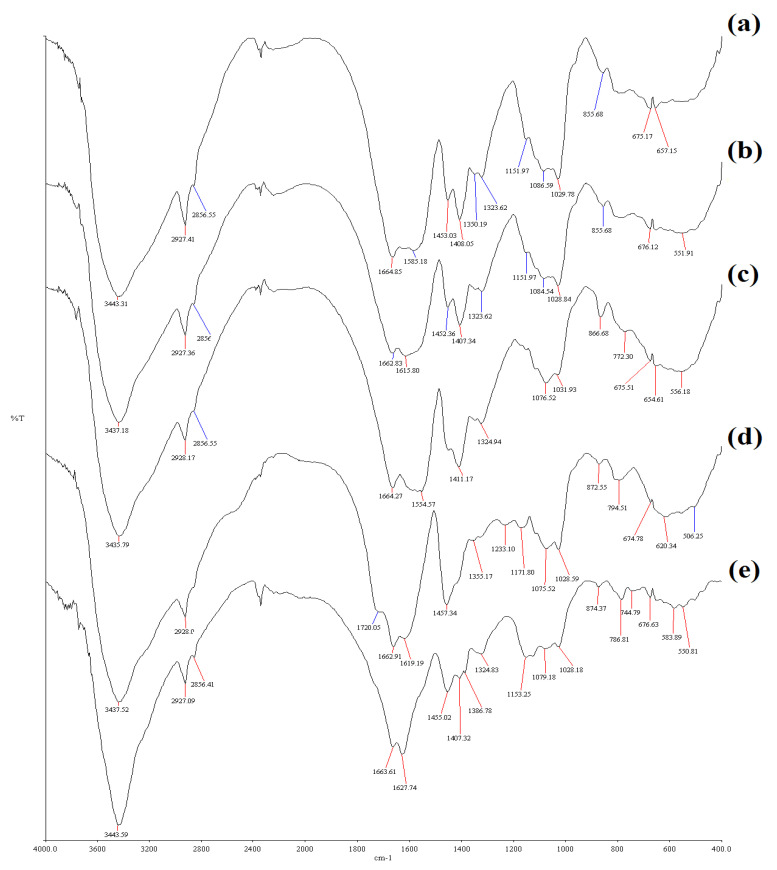
FTIR spectra (lyophilized) H-Na-PCMSA-g-PAN sample after swelling in (**a**) water with a poor conductivity; (**b**) 0.15 M NaCl solution; (**c**) 0.15 M CaCl_2_ solution; (**d**) 0.15 M AlCl_3_ solution and (**e**) 0.15 M SU solution.

The IR spectrum of the hydrogel sample swollen in water is displayed in Figure 7a. This spectrum shows that the crest of the (untreated) hydrogel which appeared at ~1663 cm^−1^ (not shown) did not change in water-treated sample. However, the absorption at ~1632 cm^−1^ shifted to ~1586 cm^−1^ in this sample, indicating a strong interaction between a water molecule and H_2_N-C=O moiety as indicated below: 

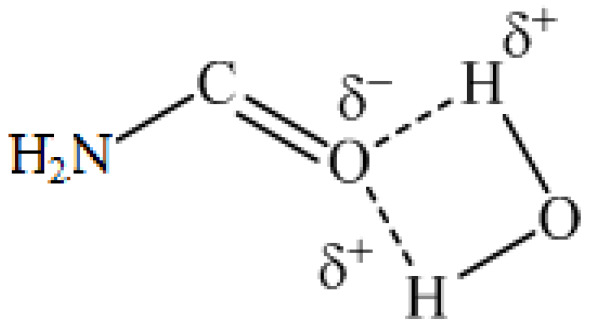



This will lead to a substantial decrease in the C=O stretching. It is further interesting to note that in the case of the hydrogel sample swollen in water, no apparent change in absorption assigned to carboxylate moiety was observed. This indicated no substantial interaction between water and the carboxylate ion.

Figure 7b represents the IR spectrum of the hydrogel sample swollen in 0.15 M NaCl solution. It is seen from this spectrum that no noticeable changes were registered in the absorptions associated with carboxylate moiety and stretching of C=O group in NH_2_-C=O. Thus, no interaction with these is envisaged. A similar observation has been reported by an earlier researcher [35].

The IR spectrum of the hydrogel sample swollen in 0.15 M CaCl_2_ solution is revealed in Figure 7c. In the IR spectrum, a broad absorption comprising of the crests at ~1600 cm^−1^ and ~1554 cm^−1^ was noticed. The absorption band which was appeared at ~1454 cm^−1^ in the hydrogel sample (not shown) was found to be affected significantly when the sample swollen in 0.15 M CaCl_2_ solution. This band was found to have practically disappeared. Furthermore the apex at ~1564cm^−1^ also lifted to ~1554 cm^−1^. These observations led to confirm that the carboxylate ion interacted significantly with Ca^2+^ ions. 

Figure 7d represents the IR spectrum of the hydrogel sample swelling in 0.15 M AlCl_3_ solution. The bands of absorption that seemed at ~1564 cm^−1^ and ~1407 cm^−1^, primarily due to carboxylate ions in the hydrogel (not shown), were absent in the hydrogel sample swollen in 0.15 M AlCl_3_ solution. This signifies strong interaction of AlCl_3_ with carboxylate ions. Surprisingly a novel apex was seen at ~1720 cm^−1^ indicating C=O stretching in −COO moiety.

The existence of this can be visualized if the coordination of the following type takes place with AlCl_3_:

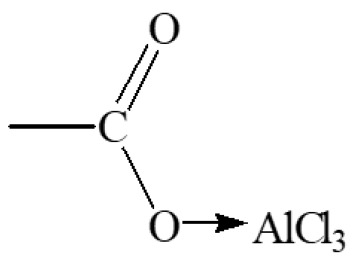



Thus, a strong interaction of Al^3+^ with carboxylate ion is proposed.

The IR spectrum of the hydrogel sample swollen in 0.15 M SU solution is shown in Figure 7e. The SU solution consists of NaCl, CaCl_2_, MgSO_4_, and Urea. The bands of the hydrogel (not shown) associated with carboxylate ion were significantly affected when the hydrogel sample was swollen in the SU solution. The peak of the hydrogel which appeared at ~1564 cm^−1^ (not shown) was found to be absent in the SU-treated sample [cf. Figure 7e]. The intensity of peak at ~1407 cm^−1^ was also found to be decreased considerably. It can be inferred from these observations that strong interactions existed between SU and the carboxylate ion of the hydrogel. It is known that CaCl_2_ will react with MgSO_4_ forming CaSO_4_ which is insoluble in water. Hence Ca^2+^ ions may not be involved to interact with the carboxylate ion. The Mg^2+^ ions will interact with carboxylate groups to shift their absorptions. It is not possible to predict other interactions as urea also has absorption at ~1675 cm^−1^ and ~1605 cm^−1^.

#### 2.4.2. Scanning Electron Microscopy (SEM)

**Figure 8:**
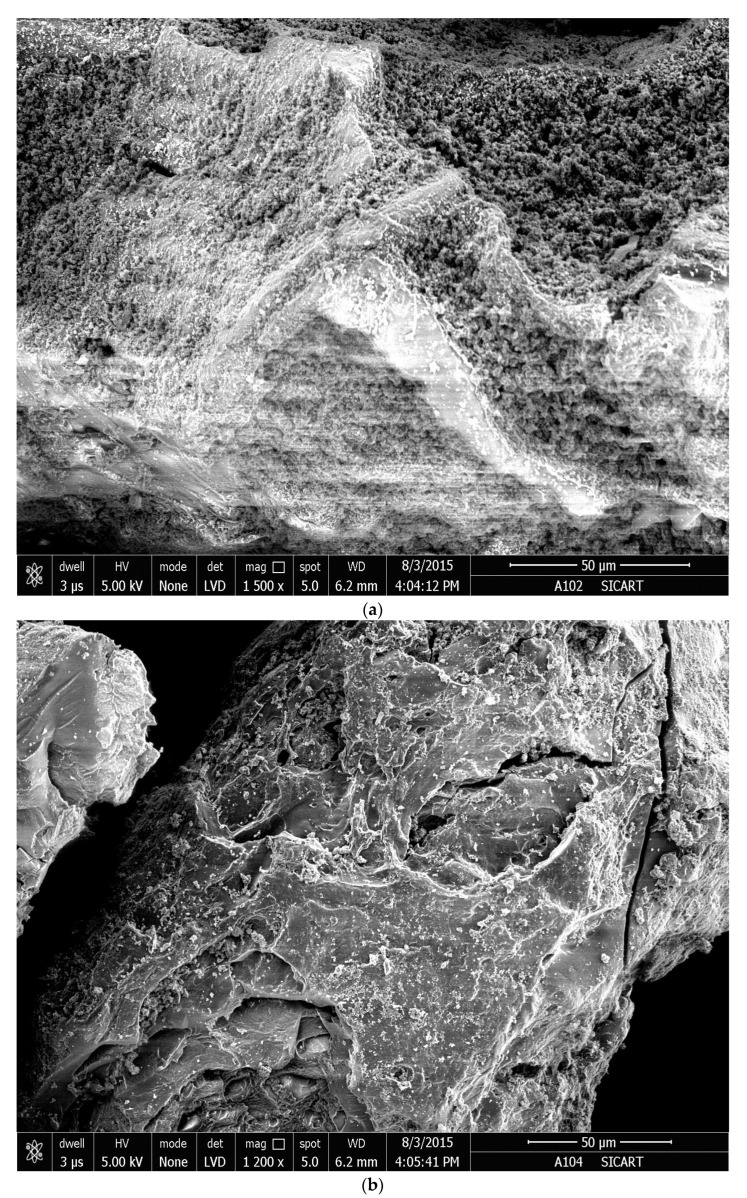

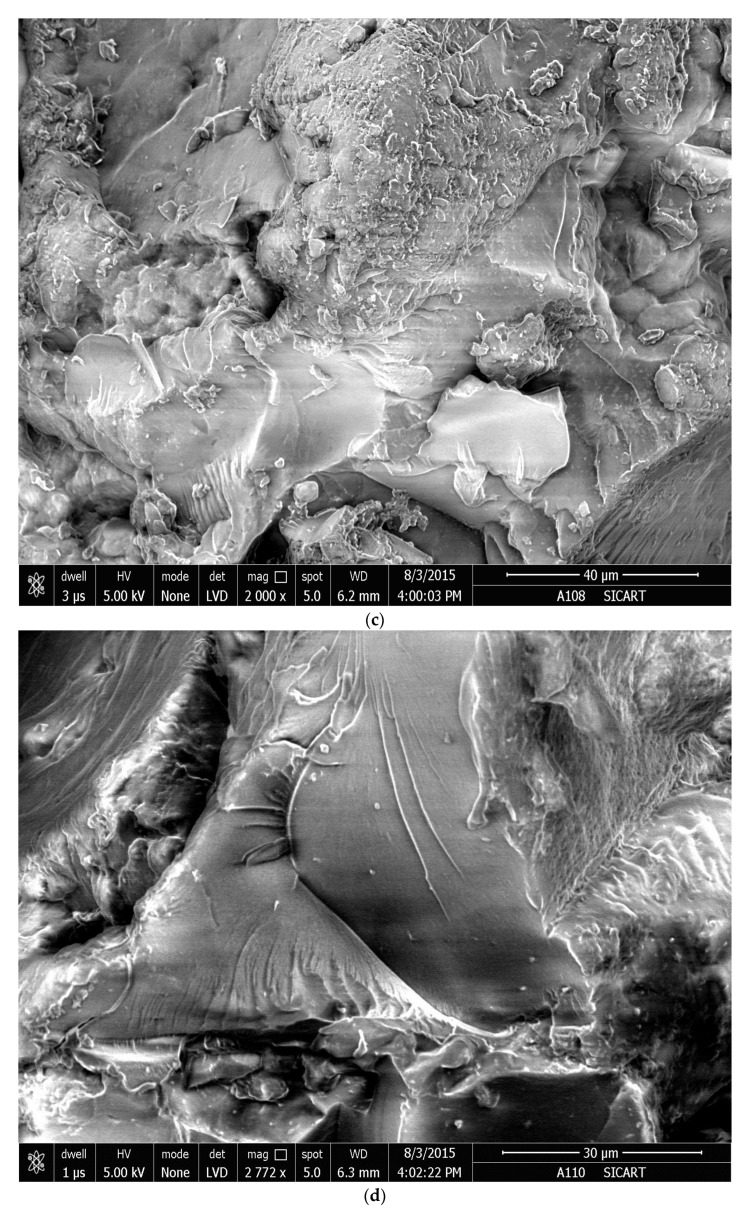

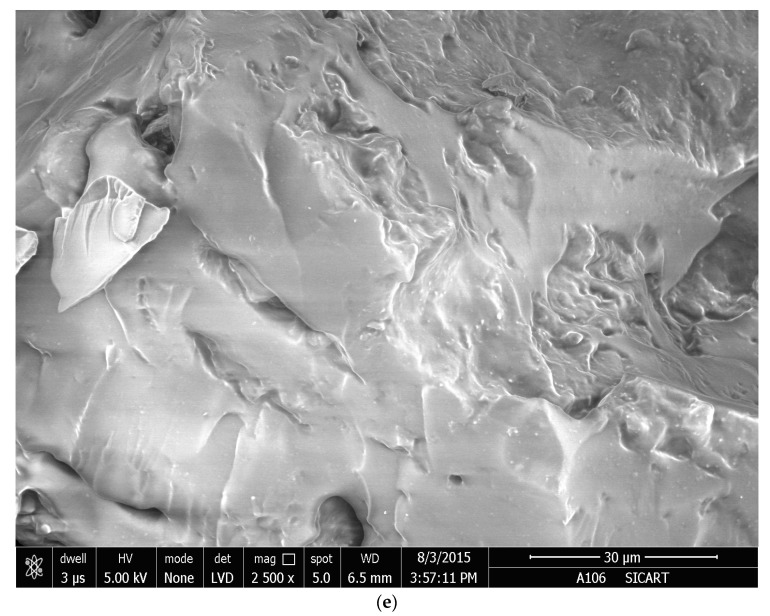
Scanning Electron Microscopy pictures of the lyophilized hydrogel samples swollen in (**a**) water with a poor conductivity; (**b**) 0.15 M NaCl solution; (**c**) 0.15 M CaCl_2_ solution; (**d**) 0.15 M AlCl_3_ solution and (**e**) 0.15 M Simulated Urine (SU).

SEM provides insight into the hydrogels’ microstructure. The cross-sections of the freeze-dried hydrogel samples are depicted in Figure 8a–e. The cross-sections of the hydrogel samples swelled in different swelling media exhibit micropores architectures. An enhanced surface area and capillary effect result from this porous microstructure [Figure 8a–e].

In the SEM micro pictures of the hydrogel samples swollen in water with a poor conductivity [Figure 8a] and 0.15 M NaCl solution [Figure 8b], the micro-scale layered porous structures are obtained. Increased surface area is a result of these layers. In water with a poor conductivity and 0.15 M NaCl solution, the fluid easily diffuses into the hydrogel mass through the gaps of the layers leading to a substantial increment of the absorption rate. The surface morphology of the hydrogel samples swollen in 0.15 M CaCl_2_ and AlCl_3_ solutions [Figure 8c,d] have rough surfaces. While the surface morphology of the hydrogel sample swollen in 0.15 M SU solution [Figure 8e] has a dense and smooth surface. The surfaces as observed in Figure 8a–e are convenient for the penetration of different saline solutions into the polymeric network. These observed morphologies are in good agreement with the results of our equilibrium water absorbency measurements.

## 3. Conclusions

In the current study, the optimally prepared photo-induced graft copolymer, Na-PCMSA-g-PAN (%G = 316.53 and %GE = 99.31) was saponified to accomplish in-situ cross-linked hydrogel network (H-Na-PCMSA-g-PAN) with very high capability of water absorption (absorbency in water with a poor conductivity, 295.31 g/g gel; absorbency in 0.15 M NaCl, CaCl_2,_ and AlCl_3_ solutions to be 96.90 g/g gel; 90.19 g/g gel and 67.68 g/g gel respectively). Additionally, in simulated urine (SU), the hydrogel showed the maximum swelling ability of the order of 93.98 g/g gel. 

The inflammation behavior of H-Na-PCMSA-g-PAN was investigated in water with a poor conductivity as well as in varied saline solutions (0.15 M) including NaCl, CaCl_2_, AlCl_3_, and SU. The hydrogel’s ability to expand in saline solutions with the same concentration (0.15 M) is found in the following order: NaCl > CaCl_2_ > AlCl_3_. The water absorption capacity of the hydrogel was also deliberated in diverse aqueous saline solutions (NaCl), and the results depicted that a decrease in water absorption capacity is observed with increasing ionic strength, indicating support for the Flory thoughts. The value of the factor f was also calculated for 0.15 M varied saline solutions, and its values were found to be higher in 0.15 M CaCl_2_, AlCl_3_, and SU solutions compared with 0.15 M NaCl solutions containing monovalent cations. Based on the “charge screening effect” and “ionic crosslinking” of the cations, the results regarding the swelling pattern of the hydrogel have been explained. 

Additionally, it was discovered that the hydrogel’s swelling process obeys pseudo-second-order-kinetics in variety of inflammation media after the experimental results regarding the hydrogel’s water absorption in variety of saline (0.15 M) solutions were analyzed in terms of the first-; and second-order kinetic models. FTIR technique has been successfully utilized not only to confirm the structure of the products but also to show the change in chemical environment to $-COO^{-}$ and −CONH_2_ groups after swelling in water with a poor conductivity and various saline solutions. The hydrogel samples have also been characterized using the SEM technique. 

The superabsorbent hydrogel’s salt sensitivity, good water absorption capacity, and water retention capacity established through the current research may be used both as a diaper and as an adsorbent material.

## 4. Materials and Methods

### 4.1. Materials

After being purified, a home-made sample of Na-PCMSA ($\bar{DS}$ = 1.10) was employed. Both sodium hydroxide (Samir Tech Chem., Baroda, Gujarat, India) and ceric ammonium nitrate (CAN, Qualigens Glaxo India, India) were used exactly as received. At atmospheric pressure, acrylonitrile (Fluka, Switzerland) was distilled off, and the middle portion was gathered and used. Urea and sodium chloride both of analytical reagent grade, were utilized as received from the Maruti Chemicals Corporation, Anand, Gujarat, India. The analytical reagent grade aluminum chloride (Loba Chemicals, Mumbai, India), as well as calcium chloride and magnesium sulphate (Samir Tech. Chem., Baroda, Gujarat, India), were employed exactly as given. The reagent grade was used for all other chemicals and solvents used in the current experiment. Nitrogen gas was filtered by transient through a brand-new pyrogallol solution. The reactions of photo-graft copolymerization and solution preparation were carried out in water with a poor conductivity.

### 4.2. Methods

#### 4.2.1. Synthesis of Na-PCMSA-g-PAN

Na-PCMSA-g-PAN (%G = 316.53, %GE = 99.31) was prepared using photo-initiated synthesis under the predetermined optimal reaction conditions: Na-PCMSA ($\bar{DS}$ = 1.10) = 0.2 g (dry basis); [CAN] = 0.05 mol/L; [HNO_3_] = 0.2 mol/L; [AN] = 0.152 mol/L; Time = 4h; Temperature = 30 °C and Total Volume = 150 mL as discussed in the first part of this study [25]. 

#### 4.2.2. Saponification or Alkaline Hydrolysis

Following the method outlined in the first part of this study [25], the graft copolymer sample, Na-PCMSA-g-PAN (%G = 316.53, %GE = 99.31), was saponified to develop the superabsorbent hydrogel-H-Na-PCMSA-g-PAN. Figure 1 shows the saponification reaction of Na-PCMSA-g-PAN to make the superabsorbent hydrogel.

#### 4.2.3. Swelling Kinetics

The swelling capacity (S) and the equilibrium water content (EWC%) of the superabsorbent hydrogel were measured using the traditional gravimetric method. A tea bag containing 0.1 g of an accurately dried powdered sample of the hydrogel, after passing through a 100 mesh (150 μ_m_) sieve, was submerged in 200 mL of water with a poor conductivity and left to soak at room temperature for varying lengths of time (0.5 h to 24 h). The balanced swollen gel was made to drain after every measurements by removing the tea bag from the water and dangling it up for 10 min or until no more droplets were drained. The weight of the swellon gel was then determined by weighing the bag. 

Similarly by following the same procedure, the absorbing measurements of the hydrogel were also carried out in 0.15 M aqueous solutions of NaCl, CaCl_2_, AlCl_3_, and Simulated Urine (SU: composition: 0.85 g CaCl_2_.2H_2_O; 1.14 g MgSO_4_.7H_2_O; 8.20 g NaCl; 20 g Urea and 1000 mL water with a poor conductivity) solution [36] for varying time intervals (0.5 h to 24 h). Additionally, by using the same process as the previously mentioned method, the hydrogel’s ability to swell in NaCl solutions of various concentrations was determined. 

Thus, by using the swelling experimental weights of the hydrogel, the value of the inflammation capacity (S) was measured thrice at room temperature in each case utilizing the formula below:

(5) $$S\;=\;\frac{W_{s}-W_{d}}{W_{s}}$$

where $W_{s}$ and $W_{d}$ are the weights of the swollen gel and the dry gel, respectively. The swelling capacity (S), thus, was calculated as grams of water per grams of hydrogel sample (g/g gel).

The equilibrium water content (EWC%) which is the inflammation capacity of the hydrogel at equilibrium was also calculated utilizing the subsequent formula:

(6) $$EWC\;(\%)\;=\;\frac{W_{eq}-W_{d}}{W_{eq}}\times 100$$

where $W_{eq}$ is the weight of the swollen gel at equilibrium.

#### 4.2.4. Instrumental Analysis

Nicolet Impact 400 D Fourier Transform Infrared Spectrophotometer was employed to document the FTIR spectra of the superabsorbent hydrogel, H-Na-PCMSA-g-PAN, freeze-dried samples (swollen in water with a poor conductivity, 0.15 M NaCl, CaCl_2_, AlCl_3_, and SU solutions), in the form of KBr pellets.

The superabsorbent hydrogel samples, at their maximum swelling capacity (in water with a poor conductivity as well as in 0.15 M NaCl, CaCl_2_, AlCl_3,_ and SU solutions) were frozen at −70 °C and then freeze-dried under vacuum for three days until all water was sublimed. Following a gold coating and scanning with an increasing voltage of 5 kV, the freeze-dried samples were carefully fractured to examine the morphology of their cross-sections under a scanning electron microscope (SEM) (Model ESEM TMP + EDAX, Philips).

## Figures and Tables

**Table 1:** Relationship between the types of swelling media employed at different times and the Dimensionless Saline Sensitivity (f) of H-Na-PCMSA-g-PAN.

Time (min)	$f_{NaCl}$	$f_{CaCl_{2}}$	$f_{AlCl_{3}}$	$f_{SU}$	Average Salt Sensitivity ^a^
15	0.625	0.656	0.678	0.643	$f_{NaCl}$ = 0.626 $f_{CaCl_{2}}$ = 0.709 $f_{AlCl_{3}}$ = 0.755 $f_{SU}$ = 0.668
30	0.679	0.724	0.682	0.717	
60	0.512	0.679	0.725	0.614	
90	0.561	0.691	0.769	0.581	
120	0.496	0.733	0.789	0.609	
150	0.532	0.718	0.748	0.618	
180	0.573	0.735	0.740	0.649	
210	0.661	0.746	0.767	0.716	
240	0.719	0.752	0.793	0.732	
360	0.680	0.714	0.761	0.705	
480	0.671	0.704	0.762	0.694	
600	0.672	0.699	0.772	0.695	
720	0.672	0.698	0.776	0.680	
960	0.672	0.694	0.777	0.688	
1440	0.670	0.698	0.783	0.683	

^a^ Calculated using Equation (2).

**Table 2:** Swelling Characteristics for H-Na-PCMSA-g-PAN in different swelling media.

Swelling Media	Experimental Equilibrium Swelling Capacity	$t_{req}^{a}$	$S_{eq}^{b}$	$EWC^{c}$	$r_{i}^{d}$	Ionic Strength ^e^	k^f^_s_ × 10^5^	R^2^
	(g water/g gel)	(h)	(g water/g gel)	(%)	[(g water/g gel) min]	(mole-ion dm^−3^)	[(g.gel/g.water)/min]	
low-conductivity water	295.31	10	526.32	99.66	1.87	---	0.673	0.974
NaCl (0.15 M)	96.90	10	106.38	98.98	1.15	0.15	10.20	0.995
CaCl_2_ (0.15 M)	90.19	16	98.04	98.90	0.63	0.45	6.55	0.995
AlCl_3_ (0.15 M)	67.68	8	71.43	98.54	0.72	0.90	14.10	0.982
Simulated Urine (SU)	93.98	12	100	98.95	0.87	0.18	8.68	0.998

Values are recorded as mean ± standard deviations. ^a.^ Time required to achieve Experimental Equilibrium Swelling Capacity value. ^b.^ Theoretical Equilibrium Swelling Capacity. ^c.^ Equilibrium Water Content. ^d.^ Initial Swelling Rate. ^e.^ $\mu \;=\;\frac{1}{2}\;\sum mz_{i}^{2}$, where µ, m and z_i_ are the ionic strength, the ionic concentration and charge on each individual ion, respectively. ^f.^ Swelling Rate Constant.

## Data Availability

Not applicable.
